# The Number or Type of Stimuli Used for Somatosensory Stimulation Affected the Modulation of Corticospinal Excitability

**DOI:** 10.3390/brainsci11111494

**Published:** 2021-11-12

**Authors:** Sho Kojima, Shota Miyaguchi, Hirotake Yokota, Kei Saito, Yasuto Inukai, Naofumi Otsuru, Hideaki Onishi

**Affiliations:** Institute for Human Movement and Medical Sciences, Niigata University of Health and Welfare, 1398 Shimami-cho, Kita-Ku, Niigata-City 950-3198, Niigata, Japan; miyaguchi@nuhw.ac.jp (S.M.); yokota@nuhw.ac.jp (H.Y.); kei-saito@nuhw.ac.jp (K.S.); inukai@nuhw.ac.jp (Y.I.); otsuru@nuhw.ac.jp (N.O.); onishi@nuhw.ac.jp (H.O.)

**Keywords:** transcranial magnetic stimulation, mechanical tactile stimulation, electrical stimulation, short-latency afferent stimulation

## Abstract

Motor evoked potentials (MEPs) evoked by transcranial magnetic stimulation (TMS) a few milliseconds after this cortical activity following electrical stimulation (ES) result in an inhibition comparable to that by TMS alone; this is called short-latency afferent inhibition (SAI). Cortical activity is observed after mechanical tactile stimulation (MS) and is affected by the number of stimuli by ES. We determined the effects of somatosensory stimulus methods and multiple conditioning stimuli on SAI in 19 participants. In experiment 1, the interstimulus intervals between the conditioning stimulation and TMS were 25, 27 and 29 ms for ES and 28, 30 and 32 ms for MS. In experiment 2, we used 1, 2, 3 and 4 conditioning stimulations of ES and MS. The interstimulus interval between the ES or MS and TMS was 27 or 30 ms, respectively. In experiment 1, MEPs were significantly decreased in both the ES and MS conditions. In experiment 2, MEPs after ES were significantly decreased in all conditions. Conversely, MEPs after MS were significantly decreased after one stimulus and increased after four stimulations, indicating the SAI according to the number of stimuli. Therefore, the somatosensory stimulus methods and multiple conditioning stimuli affected the SAI.

## 1. Introduction

Somatosensory input by peripheral stimulation affects the excitability of the primary motor cortex (M1), which is involved in movement [1,2,3,4,5,6,7,8,9]. Previous studies reported the existence of projections between M1 and the primary somatosensory cortex (S1), which is involved in the somatosensory process, and that somatosensory stimulation modulates M1 excitability [10,11]. Furthermore, sensory feedback from somatosensory input contributes to accurate movement and motor learning [12,13,14]; thus, the relationship between M1 and S1 excitability is also important from the perspective of motor control.

Cortical activity has been observed in the contralateral S1 approximately 20 ms after peripheral somatosensory input [15,16,17,18]. Furthermore, motor evoked potentials (MEPs) evoked by transcranial magnetic stimulation (TMS) immediately after this cortical activity resulted in an inhibition comparable to that evoked by single TMS alone; this inhibitory phenomenon is known as short-latency afferent inhibition (SAI). SAI has been observed approximately 20 ms after nerve stimulation, such as that of the median nerve and the digital nerve [5,8,19,20,21], and a previous study from our group reported SAI at 20–40 ms after digital nerve stimulation [5]. Bailey et al. (2016) reported that SAI after median and digital nerve stimulation fluctuates according to the intensity of the stimulus and that the inhibitory effects of afferent stimuli depend on the amplitudes of the somatosensory evoked potentials, indicating S1 excitability. They also suggested that SAI reflects the inhibitory effects on M1 excitability from SI activity after peripheral somatosensory input. In addition, we reported that cathodal transcranial direct current stimulation, which decreases the cortical excitability of the stimulus spot, over the S1 decreased the inhibitory effects of SAI [22]. Similarly, several studies reported that noninvasive brain stimulation, which modulates cortical excitability in a method-dependent manner, over the M1 or S1 modulates the SAI effects according to the stimulation site and method [22,23,24,25]. These results suggest that the variability of SAI is the result of the interrelated excitability in the M1 and S1 and is an important indicator of sensorimotor integration.

Many previous studies used mixed nerve and digital nerve stimulation as somatosensory stimulation methods to measure SAI. In an experiment using electroencephalography (EEG) and magnetoencephalography (MEG), the cortical response following somatosensory input was recorded as a clear waveform by nerve stimulation, such as median or digital nerve stimulation [15,16,17,18,26]. This is attributable to the advantage of using electrical stimulation (ES), which enables the establishment of an “on and off” stimulus and the triggering of synchronous activity in the neurons. Conversely, somatosensory stimulation via ES is an unusual stimulus, whereas that of skin receptors, such as tactile and vibratory stimulation, is often used in daily life. However, it is unclear whether these somatosensory stimulations elicit the same response as ES. Using MEG, Onishi et al. (2013) [18] reported that a cortical response similar to that observed after ES was detected after mechanical tactile stimulation (MS) using plastic pins. Thus, we hypothesized that MS evokes an SAI-like modulation. In addition, previous studies that recorded the cortical response after ES reported that the amplitude of the cortical response was affected by the number of ESs when the ES was applied at constant intervals [26,27,28]. Therefore, the modulatory effect of S1 activity on SAI is thought to depend on the number of somatosensory stimulations.

Hence, the aim of this study was to determine the effects of different somatosensory input methods and of the number of somatosensory stimulations on corticospinal excitability using ES and MS.

## 2. Materials and Methods

### 2.1. Participants

Twenty healthy volunteers were recruited in this study; however, one participant refused participation. Therefore, 19 healthy volunteers aged 20–30 years (mean ± standard deviation, 22.2 ± 2.8 years; four females, three left-handed) participated in this study. None of the participants engaged in drug use or used medication that affected the central nervous system. All participants provided written informed consent and conducted screening for TMS safety. This study was approved by the Ethics Committee of Niigata University of Health and Welfare (18157-190311) and was conducted in accordance with the Declaration of Helsinki.

### 2.2. Measurement of Corticospinal Excitability

This study used MEP as an indicator of corticospinal excitability. MEPs were recorded from the relaxed first dorsal interosseous (FDI) muscle of the right hand, using silver/silver chloride electrodes in a belly-tendon montage. Electromyogram signals were amplified 100× (A-DL-720-140 Amplifier; 4 Assist, Tokyo, Japan), digitized at 10 kHz using an A/D converter (Power Lab 8/30; AD instruments, Colorado Springs, CO, USA) and a high-pass filter at 20 Hz, and analyzed using Lab Chart 7 (AD instrument).

We used monophasic-pulse TMS to elicit MEPs. TMS was delivered to the left M1 using a figure-eight-shaped coil (diameter, 95 mm) connected to a Magstim 200 square instrument (Magstim, Dyfed, UK). The coil was held with the handle pointing backward and laterally approximately 45° to the sagittal plane. The optimal spot for eliciting MEPs was carefully determined in each participant and was defined as the point at which TMS evoked the largest MEP at the lowest stimulation intensity. The optimal coil position was marked on a cap worn by the participants. Moreover, the hot spot was displayed on a magnetic resonance image (MRI) as a reference, and the position and orientation of the coil were monitored throughout the experiment by MRI using the Visor2 TMS Neuronavigation System (eemagine Medical Imaging Solutions GmbH, Berlin, Germany). The optimal spot of the FDI muscle was recorded, with the coil manually held in place to maintain its position during the experiment. T1-weighted MRI was performed using a 1.5 T system before the experiment (Signa HD, GE Healthcare, Milwaukee, WI, USA). The TMS intensity (% maximal stimulator output (%MSO)) was set at a value that induced an average MEP with a peak-to-peak amplitude of approximately 1 mV, with monitoring to ensure the FDI muscle stayed relaxed during the measurement.

### 2.3. Conditioning Stimulation Setting

The conditioning stimuli of ES and MS were delivered to the right index finger. ES was delivered by an electrical stimulator (SEN-8203, Nihon Kohden, Tokyo, Japan) and ring electrodes at an intensity of three times the perceptual sensory thresholds, with a 0.2 ms square wave [5]. The stimulating cathode electrode was placed immediately distal to the metacarpophalangeal joint, with the anode electrode placed immediately distal to the proximal interphalangeal joint [5,19]. MS was delivered by piezoelectric actuators (TI-1101; KGS, Saitama, Japan) and four tiny plastic pins as follows: diameter, 1.3 mm; height of the protrusion, 0.8 mm and pushing force, 0.031–0.12 N/pin [18,29,30]. The distance between the pins was set at 2.4 mm. An MS with a protruding duration of 1 ms was applied to the tip of the right index finger. This device was used in a previous study [18] and was shown to clearly evoke the cortical response after stimulation.

### 2.4. Experiment 1: Effects of the Interstimulus Interval between the One Conditioning Stimulation and TMS on Corticospinal Excitability

**Figure 1:**
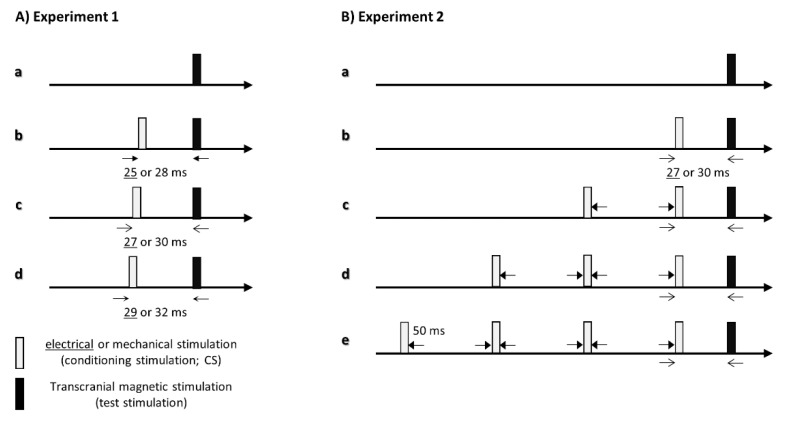
Schematic representation of somatosensory stimulation and transcranial magnetic stimulation (TMS). (**A**) Paradigm of experiment 1. Motor evoked potentials were measured by TMS alone (Aa: single), electrical stimulation conditions (Ab: ISI, 25 ms; Ac: ISI, 27 ms; Ad: ISI, 29 ms) and mechanical tactile stimulation conditions (Ab: ISI, 28 ms; Ac: ISI, 30 ms; Ad: ISI, 32 ms). (**B**) Paradigm of experiment 2. Motor evoked potentials were measured by TMS alone (Ba: single) and four different numbers of repetitions (Bb: 1 stim; Bc: 2 stim; Bd: 3 stim; Be: 4 stim) of each somatosensory stimulation; the interstimulus interval between the somatosensory stimulation and TMS was set to 27 ms (electrical stimulation) and 30 ms (mechanical tactile stimulation).

The aim of experiment 1 was to investigate the effects of the interstimulus interval between the conditioning stimulation and TMS on corticospinal excitability (Figure 1A). The interstimulus interval was based on the somatosensory evoked magnetic fields (SEFs) elicited by each somatosensory stimulation. A previous study reported that a significant first component of cortical activity immediately after electrical stimulation was recorded at an average latency of 25 ms and that a significant first component of cortical activity immediately after mechanical tactile stimulation was recorded at an average latency of 28 ms [18]. In this study, we set the interstimulus interval at this average time, average time +2 ms and average time +4 ms in experiment 1. The interstimulus interval between the conditioning stimulation and TMS was 25, 27 and 29 ms in the ES condition, and 28, 30 and 32 ms in the MS condition. Single-pulse TMS alone (single) was used as the control condition. MEP measurements were performed randomly for each of the 15 stimuli of the seven conditions (three conditions each for ES or MS, and the control condition). TMS was delivered at intervals of 5–6 s.

### 2.5. Experiment 2: Effects of the Number of Conditioning Stimuli on Corticospinal Excitability

The aim of experiment 2 was to investigate the effects of the number of conditioning stimuli on corticospinal excitability (Figure 1B). In this experiment, 1, 2, 3 and 4 conditioning stimuli were used, with an interval between each conditioning stimulus of 50 ms. The interstimulus interval was set based on the SEF recorded by MEG. The cortical peak activity following ES and MS was clearly observed at approximately 50–60 ms [18], so we set 50 ms as the interstimulus interval between each conditioning stimulus. The interstimulus interval between the conditioning stimulation and TMS was 27 ms (ES) or 30 ms (MS). MEP measurements were established as described for experiment 1, and the experiments were performed on the same day.

### 2.6. Data and Statistical Analyses

The mean MEP amplitudes were calculated from the peak-to-peak amplitudes of 13 of the 15 trials, with elimination of the largest and smallest values [5,7]. Statistical analyses were performed using SPSS statistics 24 software (IBM SPSS, Armonk, NY, USA). All MEP data (experiments 1 and 2) followed the normal distribution. The mean MEP amplitudes of experiment 1 were analyzed using two-way repeated measures analysis of variance (ANOVA) (CONDITION (ES and MS) × ISI (ES: single, ISI: 25 ms, ISI: 27 ms and ISI: 29 ms) (MS: single, ISI: 28 ms, ISI: 30 ms and ISI: 32 ms)), and the effect size of the ANOVA was calculated using the partial eta-squared (partial η^2^). The sphericity of the data was tested using Mauchly’s test, and Greenhouse–Geisser-corrected significance values were used when sphericity was lacking. Post hoc analyses to compare each MEP of the time conditions were performed using Dunnett’s tests. We calculated the MEP ratio (the MEP amplitude with conditioning stimulation/the MEP amplitude of the single stimulation) to compare the degree of MEP modulation and identify each interstimulus interval that had the lowest MEP ratio. Subsequently, a paired t-test was used to compare the MEP ratio obtained at this ISI with ES and MS. Moreover, the correlation of MEP modulation between the ES and MS condition was assessed using Pearson’s correlation analysis. The mean MEP amplitudes of experiment 2 were statistically analyzed by two-way repeated-measures ANOVA (CONDITION (ES and MS) × STIMULI (1, 2, 3 and 4 times)), and the effect size of the ANOVA was calculated using the partial η^2^. Post hoc analyses were performed using Dunnett’s tests to compare each MEP of the time conditions. Statistical significance was set at a *p*-value of <0.05.

## 3. Results

The mean intensities of TMS and ES (mean ± SD) used in this study were 56.5 ± 6.9 %MSO and 6.4 ± 2.5 mA, respectively. No patients experienced pain following ES and MS.

### 3.1. Experiment 1

**Figure 2:**
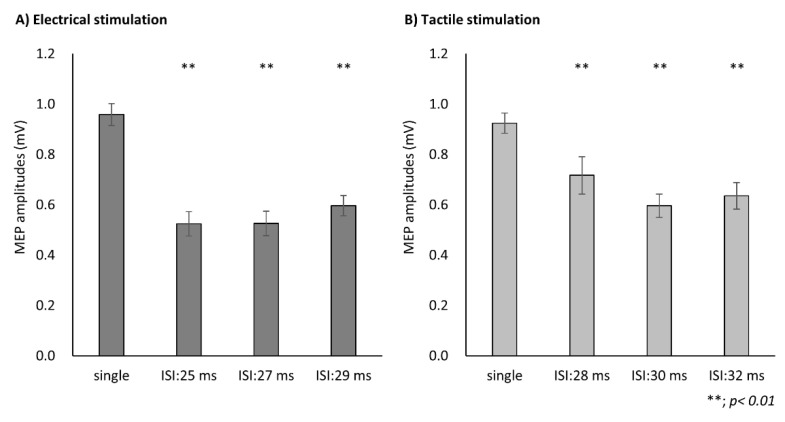
Mean motor evoked potentials (MEPs) of each condition. Correlation and comparison of the motor evoked potential (MEP) ratio after each conditioning stimulus. (**A**) Pearson’s correlation analysis revealed a significant correlation between the maximum inhibitory effects of electrical stimulation and mechanical tactile stimulation in each participant. (**B**) The inhibitory effects of MEP after electrical stimulation were significantly higher than those after mechanical tactile stimulation. Data are expressed as the mean ± standard error of the mean (SEM).

Two-way repeated measures ANOVA revealed a significant main effect of CONDITION (F(1, 18) = 2.52, *p* = 0.01, partial η^2^ = 0.12) and TIME (F(3, 54) = 49.42, *p* < 0.01, partial η^2^ = 0.73), and a significant interaction of CONDITION × ISI (F(3, 54) = 3.82, *p* = 0.02, partial η^2^ = 0.18). In ES condition, the post hoc analysis showed a significant decrease in the MEP at each ISI compared with single stimulation (ISI: 25 ms, *p* < 0.001; ISI: 27 ms, *p* < 0.001; ISI: 29 ms, *p* < 0.001) (Figure 2A). In MS condition, the post hoc analysis showed a significant decrease in the MEP at each ISI compared with single stimulation (ISI: 28 ms, *p* = 0.002; ISI: 30 ms, *p* < 0.001; ISI: 32 ms, *p* < 0.001) (Figure 2B).

**Figure 3:**
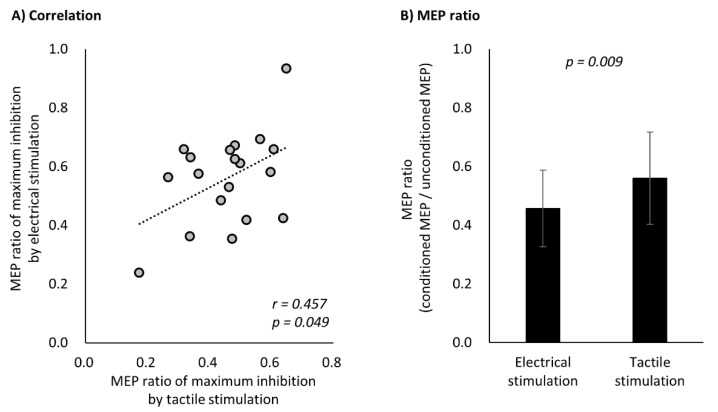
Correlation and comparison of the motor evoked potential (MEP) ratio after each conditioning stimulus. (**A**) Pearson’s correlation analysis revealed a significant correlation between the maximum inhibitory effects of electrical stimulation and mechanical tactile stimulation in each participant. (**B**) The inhibitory effects of MEP after electrical stimulation were significantly higher than those after mechanical tactile stimulation. Data are expressed as the mean ± standard error of the mean (SEM).

The lowest MEP ratio was significantly correlated with ES and MS, as assessed using Pearson’s correlation test (r = 0.457, *p* = 0.049) (Figure 3A). These MEP ratios were 0.46 ± 0.03 and 0.56 ± 0.04 with the ES and MS conditions, respectively. A paired t-test showed a significantly smaller mean MEP ratio in the ES condition than in the MS condition (*p* = 0.009) (Figure 3B).

### 3.2. Experiment 2

**Figure 4:**
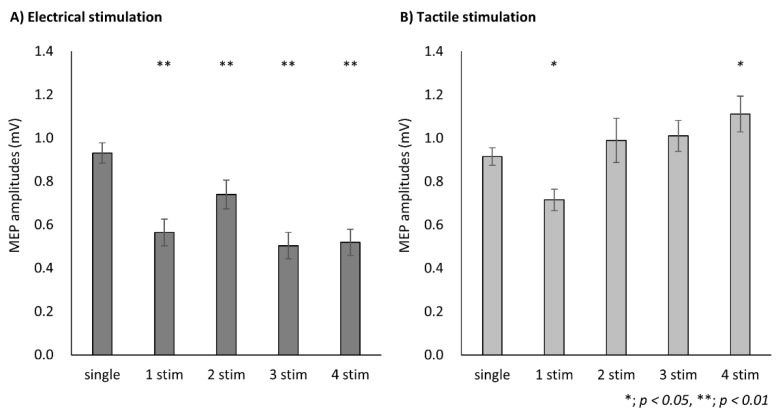
Modulation of motor evoked potentials (MEPs) according to the number of stimuli in each conditioning. Data are expressed as the mean ± standard error of the mean (SEM). (**A**) Modulation of MEP amplitudes with each number of electrical stimulations. The post hoc analysis showed a significant decrease in MEP in the presence of conditioning stimulation compared with that in the presence of single stimulation (1 stim, *p* < 0.001; 2 stim, *p* = 0.001; 3 stim, *p* < 0.001; 4 stim, *p* < 0.001). (**B**) The post hoc analysis showed a significant decrease in MEP at the conditioning stimulation of 1 stim compared with that at single stimulation (*p* = 0.030) and a significant increase in MEP at 4 stim (*p* = 0.015).

Two-way repeated-measures ANOVA revealed a significant main effect of CONDITION (F (1, 18) = 32.57, *p* < 0.01, partial η^2^ = 0.64)and STIMULI (F (4, 72) = 9.72, *p* < 0.01, partial η^2^ = 0.35), and a significant interaction (F (2.64, 47.55) = 22.53, *p* < 0.01, partial η^2^ = 0.56). In ES condition, the post hoc analysis showed a significant decrease in MEP in the presence of conditioning stimulation compared with that in the presence of single stimulation (1 stim, *p* < 0.001; 2 stim, *p* = 0.001; 3 stim, *p* < 0.001; 4 stim, *p* < 0.001) (Figure 4A). In MS condition, the post hoc analysis showed a significant decrease in MEP at the conditioning stimulation of 1 stim compared with that at single stimulation (*p* = 0.030) and a significant increase in MEP at 4 stim (*p* = 0.015) (Figure 4B).

## 4. Discussion

In this study, we investigated the effects of various somatosensory stimulus methods and multiple conditioning stimuli on corticospinal excitability using ES and MS. The results showed that the somatosensory input methods and the number of somatosensory stimulations affected the modulation of corticospinal excitability. MEP amplitudes were significantly decreased in both the ES and MS somatosensory input types, whereas the degree of MEP depression was higher with ES. Additionally, MEP amplitudes after ES were significantly decreased in the presence of conditioning stimulations (1–4 stim), indicating a decrease in the corticospinal excitability. Conversely, MEP amplitudes after MS were significantly decreased at one MS stimulation, whereas a significant increase was observed for four MS stimulations, indicating that the modulation of corticospinal excitability depended on the number of stimuli applied.

### 4.1. Effects of Different Somatosensory Input Methods on Corticospinal Excitability

In experiment 1, we investigated the effects of two somatosensory input types on corticospinal excitability. The MEP amplitudes after conditioning by both ES and MS were significantly decreased compared with that of the single TMS, indicating a decrease in corticospinal excitability. A decrease in MEP amplitude immediately after ES, such as mixed nerve and digital nerve stimulation, has been reported as SAI in many previous studies [26,31,32,33,34,35,36], with SAI depending on the stimulus intensity and the S1 activity following ES [8,37]. Pharmacological studies on the SAI mechanism reported that SAI involves GABAA receptors, cholinergic neurons, dopamine and noradrenaline, suggesting that the activity of these neurons and the transmitter induces a depression in M1 excitability [31,32,38]. In this study, a decrease in MEP amplitude was observed immediately after MS, as well as after digital nerve stimulation [2,37,39,40,41,42]; we believe that this phenomenon might be due to the presence of an SAI-like inhibitory effect after MS. A previous study reported that the S1 activity observed after MS was similar to that observed after ES [18]. Therefore, it is suggested that SAI-like inhibition is also observed after the MS-based induction of S1 activity, as well as after ES.

Furthermore, we detected a correlation between the inhibitory effects of ES and MS. The cortical activity observed following somatosensory input, such as ES and MS, was recorded by EEG and MEG, with the first response confirmed at approximately 20–30 ms after somatosensory stimulation (ES: approximately 20 ms, MS: approximately 28 ms). Moreover, this first response has been reported to reflect the S1 activity [15,16,17,18,43,44]. Considering the type of stimulation used in this study, this cortical response after somatosensory stimulation is caused by the reception of sensory information from the digital nerve in the ES or from mechanoreceptors in the MS condition. The tactile information afforded by MS is received by mechanoreceptors of the slow adaptation (SA) and rapid adaptation (RA) types, and is ultimately input to the S1 via the digital nerve [45]. Therefore, we found a significant positive correlation between the inhibitory effects of ES and MS, likely via the same pathway to the S1, although the type of stimulation was different.

Conversely, the degree of inhibition observed immediately after ES was higher than that detected immediately after MS. This result is thought to involve the S1 activity evoked by each somatosensory stimulation. Previous studies reported that the inhibitory effect of SAI increased with increasing ES intensity [37]; furthermore, the degree of the inhibitory effect was correlated with the somatosensory evoked potentials recorded by EEG after ES [8]. In this study, the mean ES intensity and MS were approximately 6.4 mA and four stimuli pins, respectively. The SEFs, which indicate S1 activity, recorded by MEG had similar waveforms after ES and MS; however, the peak strength of SEFs was reported to be greater after an ES of 6 mA vs. an MS of four stimulus pins [18]. Moreover, an early peak of SEF, as a reference of the current stimulus interval, was observed in 6 of 12 subjects after MS and in 11 of 12 subjects after ES [18]; thus, the synchronous activity of S1 was more pronounced by ES than by MS. Therefore, considering the type of somatosensory stimulation used in this study, it is suggested that ES induced more synchronous activity in the S1 than MS and that the inhibitory effects of ES with higher S1 activity were higher than those of MS.

### 4.2. Effects of the Number of Somatosensory Stimulations on Corticospinal Excitability

In experiment 2, we investigated the effects of the number of somatosensory stimulations on the corticospinal excitability. The MEP amplitudes after conditioning by 1–4 electrical stimulations were significantly smaller than that of the single TMS alone, indicating a decrease in corticospinal excitability. Ruddy et al. (2016) [46] reported that MEP amplitudes were decreased by a conditioning ES repeated three times at intervals of 3.4 ms. Many previous studies have shown that conditioning stimulation using a single ES decreases corticospinal excitability [2,5,19,20], whereas multiple conditioning stimulations were reported in a few studies. In contrast, the N20m amplitude of SEF, which indicated the S1 activity, yielded no significant changes after 1–5 or 1–6 repeated pulses of ES [26,28]. Thus, the results of this study, similar to those reported by Ruddy et al. (2016) [46], suggest that the inhibitory effects of MEP after each conditioning ES, based on the S1 activity, were similar to those observed after a single ES. Furthermore, the inhibitory effects of multiple-stimulus conditioning ES were similar to those observed for one electrical stimulus. Previous studies reported a significant increase in MEP amplitudes evoked 50–80 ms after ES compared with that by a single TMS [5,20,21,47,48]; this phenomenon is known as afferent facilitation (AF). It has been reported that AF is also evoked by digital nerve stimulation [20] and that the evoked latency varies among subjects [5]. Moreover, previous studies noted a significant decrease in MEP amplitudes evoked 100–200 ms after ES compared with that evoked by a single TMS; this phenomenon is known as long-latency afferent inhibition (LAI) [49,50]. The interval between the first ES and the TMS was set to 77 ms, 127 ms or 177 ms at the 2 stimulus, 3 stimulus or 4 stimulus ES condition, respectively. Therefore, the SAI, LAI and AF evoked by the interstimulus interval of each stimulus are thought to be involved in the MEP fluctuations during multiple-stimulus conditioning, and it was suggested that the summation of these effects was similar to that of the one-stimulus condition.

In contrast, MEP amplitudes detected after conditioning by one- or four-stimulus conditioning MS were significantly smaller or larger than that of the single TMS, respectively. These results were different from those obtained for the ES condition, and this difference in MEP modulation is thought to be related to the activity of peripheral sensory receptors. A previous study did not investigate the modulation of MEP after MS, whereas it has been reported that a significant increase in MEP amplitude was observed after 200 ms of air-puff stimulation, which pressed the skin as in the present study, compared with the MEP evoked by a single TMS alone [51]. This indicates that mechanoreceptors such as SA and RA respond to MS and increase MEP amplitudes approximately 200 ms after the afferent input from the mechanoreceptor, and that this effect of MS was different from that of ES, which yielded an inhibitory effect. In this study, the interval between the first MS and the TMS was set to 180 ms in the four-stimulus MS condition, suggesting that the first stimulation of the four-stimulus MS condition may have induced the same effect on MEP as did the air-puff stimulation. Moreover, MEP amplitudes are reported to indicate the sum of excitatory and inhibitory responses [52], as shown in the ES condition. Therefore, the results of the MS condition suggest that MEP modulation reflects the sum of the excitatory and inhibitory effects of SAI, AF and LAI by each conditioned mechanical stimulus. However, the MEP modulation over time after MS and the cortical activity level after multiple MS has not been clarified; therefore, it will be necessary to investigate the effects of the interstimulus interval between MS and TMS on MEP amplitude and the cortical activity after multiple MS in the future.

This study has several limitations. First, the individual somatosensory evoked potentials (SEPs) and SEF after ES and MS were not measured, so the difference in individual latency of SEP or SEF may affect the MEP modulation after ES or MS. However, we set the interstimulus interval between the conditioning and test stimuli based on the average latency of SEF evoked by ES and MS with the same device used in a previous study. Therefore, the influence is considered to be minimal. The second limitation is that the cortical activity of S1 after multiple ES and MS was not measured. The modulation of MEP was related to the S1 activity of somatosensory stimulation, with further investigation required into the change in S1 activity after multiple ES and MS.

## 5. Conclusions

This study showed that the somatosensory input methods and the number of somatosensory stimulations affected the modulation of corticospinal excitability. The SAI observed after ES was similar to that observed after MS. Conversely, the effect of the number of somatosensory stimulations was dependent on the stimulus method, which likely occurred via different mechanisms. Therefore, these results show the common and different effects of somatosensory stimulation methods on corticospinal excitability. These findings will contribute to a better understanding of the differences in cortical responses to tactile and electrical stimulation and the possibility of applying these results to the development of tools and the rehabilitation of patients with sensory disorders in the future.

## Data Availability

The datasets generated during and/or analyzed during the current study are available from the corresponding author.
